# The use of CAMS and DBT to effectively treat patients who are suicidal

**DOI:** 10.3389/fpsyt.2024.1354430

**Published:** 2024-07-18

**Authors:** David A. Jobes, Shireen L. Rizvi

**Affiliations:** ^1^ Department of Psychology, The Catholic University of America, Washington, DC, United States; ^2^ Graduate School of Applied and Professional Psychology, Rutgers University, Piscataway, NJ, United States

**Keywords:** treatment, suicidal ideation, suicidal behaviors, CAMS, DBT

## Abstract

Around the world, suicide ideation, attempts, and deaths pose a major public and mental health challenge for patients (and their loved ones). Accordingly, there is a clear need for effective clinical treatments that reliably reduce suicidal thoughts and behaviors. In this article, we review the Collaborative Assessment and Management of Suicidality (CAMS) and Dialectical Behavior Therapy (DBT), two clinical treatments that rise to the highest levels of empirical rigor. Both CAMS and DBT are now supported by randomized controlled trials (RCTs), with independent replications, and meta-analyses. There are also supportive data related to training clinical providers to use CAMS and DBT with adherence. RCTs that investigate the use of both interventions within clinical trial research designs and the increasing use of these complementary approaches within routine clinical practice are discussed. Future directions for research and clinical use of CAMS and DBT are explored as means to effectively treat suicidal risk.

## Introduction

Suicide is a worldwide public health challenge with over 700,000 deaths per year. Suicide rates around the world are fairly comparable with the highest rates in Africa and the lowest rates in Eastern Mediterranean countries (1). In the United States, suicide is the 11th leading cause of death with over 49,000 Americans dying by suicide and an additional 2,553,000 attempting suicide in 2022 (2). Moreover, an additional 16,600,000 American adults and teenagers struggled with *serious thoughts of suicide* in 2022. By definition, suicidal ideation is present for any self-inflicted death to be certified as a suicide, but only a scant fraction of those with suicide ideation ever go on to make suicide attempts or die by suicide. With the exception of a brief reduction in 2019 and 2020, suicides in the U.S. have steadily increased over the past five decades, while other causes of death have steadily decreased (e.g., infant mortality, influenza, tuberculosis, and HIV). The modal suicide death in the U.S. is a middle-aged white male who ends his life with a gun. The modal suicide attempter is a young adult female who overdoses. In recent years, there have been increases in suicide attempts among female adolescents and more generally among young people of color in the U.S (2). The emotional toll of suicide is profound as up to 30 “suicide loss survivors” are impacted by each suicide (3). The economic costs of suicide are considerable; in the U.S., suicide and non-fatal self-harm in 2020 cost the nation over $500 billion in medical costs, work loss costs, value of statistical life, and quality of life costs (4).

For decades, the standard clinical response for someone with suicidal thoughts and/or behaviors has been an inpatient admission to a psychiatric unit and the routine prescribing of psychotropic medications. Ironically, neither of these common clinical interventions have clear or robust empirical support. Indeed, there is evidence that inpatient hospital stays can actually be non-therapeutic (5, 6) [with a possible exception for inpatients who made a suicide attempt immediately prior to their admission (7)] and the efficacy of medications for suicidality is modest, mixed, and can even increase the risk of suicidal behaviors (8).

Fortunately, years of ongoing rigorous randomized controlled trial (RCT) clinical research has seen the development and support of a handful of psychological treatments with proven treatment efficacy and effectiveness for reducing suicidal ideation and behaviors in patients who are suicidal across different samples and clinical settings. Within any clinical trial research, the highest standard level of experimental rigor establishes a *causal* impact of a treatment (i.e., that using a treatment reliably addresses conditions reducing targeted symptoms and behaviors, leading to clinical recovery). Empirical validation thus includes the following criteria: 1) treatment studies that employ randomized controlled trial experimental designs, 2) the reliable replication of similarly supportive RCT findings, c) the replication of supportive RCT findings by *independent* researchers (i.e., investigators who did not develop the intervention with no publication bias or allegiance effects), and d) reliable empirical support of clinical trial treatment findings by at least one meta-analysis. These criteria will serve as an organizing focus for the clinical trial research studies highlighted in the present discussion.

When this level of scientific rigor is considered in relation to suicidal risk, two clinical interventions rise to the top as the most empirically supported treatments for suicidal ideation and behavior. The first treatment we will consider is the Collaborative Assessment and Management of Suicidality (CAMS) developed by Jobes (9–11). The second treatment we will discuss is Dialectical Behavior Therapy (DBT) developed by Linehan (12). While CAMS and DBT are clinically compatible, they are distinctly different psychological treatments. CAMS is provided as an individual psychotherapy modality wherein different interventions are used within ongoing care to target patient-defined “drivers” of suicide (i.e., those issues that make the patient suicidal) over 4-12 sessions of care. In contrast, comprehensive DBT is a team-based treatment emphasizing four modes of treatment delivery: 1) skills training, 2) individual psychotherapy, 3) phone coaching, and 4) team consultation. These modes are in the service of helping patients develop “a life worth living” within a behaviorally-focused treatment with care generally ranging from 6 months to more than a year. It is important to note the complementarity of these two proven treatments because they straddle the full spectrum of suicidal risk, from patients with acute suicidal ideation to patients who are chronically suicidal and have a history of multiple suicide attempts.

Both of these clinical interventions are supported by published non-randomized and randomized clinical trials as well as meta-analyses confirming their efficacy and effectiveness. To this end, based on extensive and rigorous clinical trial data, CAMS is one of the best treatments for reliably reducing suicidal ideation and overall symptom distress while increasing hope/decreasing hopelessness among outpatients and inpatients who are suicidal. In turn, there are *dozens* of RCTs as well as meta-analyses that clearly show the efficacy of DBT with data that are specific to its ability to reliably reduce suicide attempts and self-harm behaviors.

The goal of the present discussion is to review and highlight key empirical support for each intervention before turning our attention to how these treatments have increasingly been used together in clinical trial studies and also within contemporary clinical practice for reliably decreasing suicidal ideation, self-harm, and suicide attempts (among other positive secondary outcomes). Importantly, these proven clinical interventions can be provided on an outpatient basis which can obviate the need for costly emergency department (ED) and inpatient care that too often may be ineffective and are quite expensive. Moreover, effective training models for both interventions are available around the world that can help ensure their adherent clinical use for patients who suffer from suicidal thoughts and self-destructive behaviors.

Before delving into the clinical trial highlights supporting these approaches, it is important to note the outcome variables used in randomized controlled trials for suicidality can vary widely. Most studies focus on suicidal ideation and behaviors as primary outcome variables. Secondary variables may include overall symptom distress, patient satisfaction, retention to care, hopelessness, clinician satisfaction/confidence, and other “markers” related to suicide risk and treatment (e.g., depression, treatment recidivism, and cost-effectiveness). In our integrative review, we highlight the key clinical trial investigations and endeavor to report on similar outcome variables across clinical trials of CAMS and DBT.

## The Collaborative Assessment and Management of Suicidality

CAMS is an evidence-based suicide-focused therapeutic framework (11). Central to CAMS is the use of a multipurpose assessment, stabilization, treatment planning, and clinical outcome tool called the “Suicide Status Form” (SSF) that provides structured guidance and extensive clinical documentation for all CAMS suicide-focused assessment and treatment planning. There are three distinct phases to CAMS: a) the all-important first session, b) an interim phase of suicide-focused care, and c) the final outcome disposition session of CAMS. In the first session of “standard” CAMS, clinicians ask permission to take a seat next to the patient to have the patient complete an initial quantitative and qualitative assessment pertaining to the patient’s suicidality. Still sitting side-by-side, the clinician takes over assessing key risk variables and warning signs. This assessment process then leads to a collaboratively developed treatment plan that is designed to keep a patient out of the hospital, if possible. This is accomplished by collaboratively completing the CAMS Stabilization Plan (13) before shifting attention to two patient-identified problems—or “drivers”—that compel the patient to consider suicide which will be targeted and treated across the course of CAMS. Drivers identified in CAMS RCTs focus on relationships, intrasubjective misery, vocational concerns, and self-oriented issues (14). After the first session, an interim version of the SSF is used which begins with the patient rating the “SSF Core Assessment” (psychological pain, stress, agitation, hopelessness, self-hate, and overall behavior risk of suicide). All interim CAMS sessions end with an update of the CAMS Treatment Plan. For example, is the CAMS Stabilization Plan working or does it require modification? Have patient-identified suicidal drivers evolved or changed since the previous session? Is there a need to modify the driver-focused interventions? Treatment planning within CAMS is thus always suicide-focused with an overarching goal that centers on the patient learning to reliably manage their suicidal thoughts and feelings while establishing behavioral stability for three consecutive sessions. When these resolution criteria are met, there is an outcome disposition version of the SSF that is used in the final session of CAMS which brings the treatment to a close.

Beyond this general overview of CAMS, it is important to note that every session of CAMS begins with the SSF Core Assessment and ends with an update to the CAMS Treatment Plan. CAMS clinicians are free to use whatever interventions they deem appropriate to treat patient-identified drivers (CBT, behavioral activation, medication, couple’s therapy, elements of DBT or ACT, insight-oriented therapy, etc.). Patients always receive copies of their SSFs after each session over the course of care. CAMS can be administered in person or via telehealth using fillable PDF versions of the SSF (11, 15). While CAMS is typically an outpatient-oriented intervention, there has been notable success using the model in inpatient settings as well (16–19).

## Empirical support for CAMS

What follows is a review of seven randomized controlled trials of CAMS, two meta-analytic studies, and training-related research. As discussed by Jobes (11), there are 11 published non-randomized controlled trials of CAMS that consistently show positive correlational outcomes. Within the present review, we will focus on the *causal* impact of CAMS based on RCT findings (refer to **Table 1** for the included CAMS RCTs).

**Table 1:** The included CAMS RCTs.

Authors	*N*	Sample/setting	Study inclusion	Main outcomes
Comtois et al., 2011 (20)	32	CMH outpatients, Harborview, Seattle, WA	Significant SI (i.e., >13 on BSSI-C), recent SA, or imminent risk	Reduced SI and symptom distress, increased hope, patients preferred CAMS
Andreasson et al., 2016 (21)	108	CMH outpatients, Copenhagen, DK	≥2 BPD criteria, SA in the past 5 years	Mixed findings: CAMS was as effective as DBT for NSSI and SAs
Jobes et al., 2017 (22)	148	Soldier outpatients, Ft. Stewart, GA	Significant SI (i.e., >13 on BSSI-C)	Reduced SI in six to eight sessions; moderator findings: resiliency, symptom distress, decreased ED visits; cost-effective
Ryberg et al., 2019 (18)	78	Inpatients/outpatients, Oslo, NO	Significant SI (>13 on BSSI-C), positive response on questions of current SI	Reduced SI and symptom distress; moderator finding: CAMS improves poor working alliance
Pistorello et al., 2021 (23)	62	College student outpatients, University of Nevada, Reno	Rating ≥2 on question “I have thoughts of ending my life” (on a 0–4 scale)	Reductions in SI and depression; moderator finding: reductions in hopelessness
Comtois et al., 2022 (24)	150	CMH outpatients (SME)	≥1 lifetime, actual, or interrupted SA for recently discharged inpatients	Mixed findings: TAU worked better early, CAMS worked better later in terms of SI and symptom distress; clinicians were more satisfied with CAMS
Santel et al., 2023 (19)	88	Psychiatric inpatients, Bielefeld University, GE	Admitted for acute SI or recent SA	Decreased SI, symptom distress, and SA’s post D/C; stronger alliance

### Randomized controlled trials of CAMS

The published RCTs supporting CAMS provide both replicated experimental results but also independently validated results by investigations that did not include the developer (DAJ). Independent validation of RCTs means that there is no “publication bias” or “allegiance effects” that may occur when a treatment developer is involved in a clinical trial.

#### The next-day appointment RCT

The first RCT of CAMS was a small feasibility study comparing CAMS to “enhanced care as usual” (E-CAU) with 32 outpatients who were suicidal (20). Despite limited statistical power, there were significant between-group experimental findings on the primary assessment measure of suicidal ideation and secondary measures of overall symptom distress and optimism/hope. Patients receiving CAMS reported significantly higher satisfaction ratings than patients in E-CAU, and CAMS patients had significantly better treatment retention in comparison to patients receiving control care.

#### The DiaS RCT

A second CAMS RCT was conducted by researchers in Copenhagen, Denmark. The “DiaS” trial was a parallel-group superiority design in which 108 patients who attempted suicide with borderline personality disorder features were randomly assigned to either 16 weeks of DBT or up to 16 weeks of CAMS (21). This RCT found no statistically significant between-group differences between DBT and CAMS for the primary outcome variables of self-harm and suicide attempts at 28 weeks of follow-up (21). This RCT was underpowered, and while data were favorable toward CAMS, the differences did not reach statistical significance.

#### Norwegian RCT

A Norwegian research team conducted an independent RCT of CAMS (18) comparing 78 patients who were randomized to CAMS versus treatment as usual (TAU) who were recruited from seven inpatient and outpatient clinical settings (i.e., standard inpatient settings, crisis clinics, and outpatient settings). CAMS had a significantly better impact on the primary outcome variable of suicidal ideation and on the secondary outcome of overall symptom distress at 3 months of follow-up, and sustained reduction in overall symptom distress was seen among patients receiving CAMS in comparison to TAU at 12 months of follow-up (18).

#### Operation Worth Living RCT

The “Operation Worth Living” (OWL) RCT randomized 148 U.S. Infantry Army Soldiers with high levels of suicidal ideation to CAMS or E-CAU (22). At a year of follow-up, Soldiers receiving CAMS reduced their suicidal ideation (in 6-8 sessions) significantly more quickly than E-CAU (primary outcome). Soldiers in both arms of the trial generally improved on all assessment measures at the 12-month follow-up. Within a secondary analysis of possible moderators, CAMS was found to be superior to E-CAU on six of eight significant moderator findings (25). For example, married Soldiers who received CAMS had significantly more resiliency and significantly less symptom distress than Soldiers in E-CAU. There have been additional extensive secondary analyses using OWL RCT data leading to some valuable contributions [e.g (14, 26–31)].

#### SMART design study

A small clinical trial feasibility study was funded by the National Institute of Mental Health (NIMH) for an investigation that was conducted at the University of Nevada–Reno Counseling Center. This study was funded to explore the prospect of conducting a “sequential multiple assignment, randomized trial”—otherwise known as a “SMART” research design (32). The idea of a SMART is that clients can be initially randomized to one of two treatments in stage 1, which in this study meant counseling center clients would receive up to eight sessions of CAMS or TAU. Clients who insufficiently responded to stage 1 care were randomized once again in stage 2, in which they received up to 16 sessions of CAMS or DBT. The beauty of this elegant design is that researchers can investigate both within- and between-group effects along with sequencing of clinical care, the dosing of care, and how different treatments may have a differential impact on certain subtypes of clients. In this way, a SMART can help us understand for whom different treatments, at different doses, will be best suited to achieve optimal treatment outcomes.

Within the feasibility RCT, 62 counseling center students with serious suicidal ideation were randomly assigned to stage 1 care receiving either CAMS or TAU (23). Most of these students responded positively to both arms of care, and relatively few needed to be randomized to stage 2 (and this sample was too small to analyze). However, for stage 1 care, it was observed that CAMS was significantly better than TAU for reducing the primary outcome variable of suicidal ideation and a secondary outcome variable of depression. Moderator analyses from stage 1 were noteworthy since clients with no multiple attempt history or borderline features had significant reductions in a secondary variable of hopelessness when receiving CAMS when compared to TAU care. In other words, those clients with multiple suicide attempts and borderline features actually responded better to TAU care. It is important to note that all study treatments were provided by the *same clinicians* rendering whichever treatment their client had been randomly assigned to receive (either CAMS or TAU in stage 1; CAMS or DBT in stage 2). In other words, clinicians served as their own controls as their clients received different treatments provided by the exact same clinician (i.e., between-group variance that comes with different clinicians providing treatments was thus eliminated). Moreover, digital recordings of sessions were watched (using the CAMS Rating Scale to assess fidelity) which ensured that study treatments were faithfully provided as randomly assigned (23). Secondary analysis using data from this RCT showed that better adherence to the CAMS model resulted in better treatment outcomes (33).

#### Aftercare Focus Study RCT

A sixth RCT of CAMS had mixed results. The “Aftercare Focus Study” was designed to intentionally recruit extremely high-risk people—namely, patients who had made suicide attempts and were recently discharged from inpatient psychiatric care (24). In the study, 150 patients were randomly assigned to CAMS or TAU. As seen in other RCTs of CAMS, the control comparison care was actually quite strong, which perhaps eliminated some potential between-group findings. Patients in both arms of the treatment improved from baseline to 12 months of follow-up. For the primary outcome variable of suicidal ideation, there was a decrease for TAU patients at 3 months, whereas CAMS had more impact on suicidal ideation at 12 months. In addition, on a secondary outcome variable, CAMS patients had less psychological distress at 12 months when compared to baseline. One between-group secondary finding of note was that CAMS clinicians were significantly more satisfied with their treatment than TAU clinicians. The study also provided persuasive support for the notion of having outpatient clinics that specialize in clinical care that specifically focuses on suicidal risk, similar to suicide-focused clinics that exist across geographic regions in Denmark (34).

#### Inpatient CAMS RCT in Germany

Finally, a seventh published RCT of CAMS by Santel and colleagues (19) was conducted as a feasibility RCT comparing the inpatient use of CAMS to enhanced treatment as usual (E-TAU). The RCT included 88 inpatients who were acutely suicidal and admitted to a psychiatric inpatient hospital setting in Bielefeld, Germany. Results showed that both groups improved over time across all primary and secondary outcome measures. Patients receiving CAMS showed notably larger effect sizes across all measures. For treatment completers, CAMS patients showed significant improvement on the primary outcome measure of suicidal ideation (*p* = 0.01) in comparison to control patients; CAMS patients also rated the therapeutic relationship significantly better (*p* < 0.02) than E-TAU patients as a secondary outcome. Importantly, in terms of another primary outcome measure, patients treated by CAMS were significantly less likely to make a suicide attempt compared to control patients within the high-risk post-discharge period 4 weeks after they were discharged (*p* = 0.05). While encouraging, this preliminary finding needs to be further studied and replicated. Thus, within this inpatient feasibility RCT, the pattern of results was generally supportive of CAMS, suggesting that the inpatient use of CAMS is both feasible and promising. However, given the limited sample size, these preliminary findings nevertheless require further replication ideally within well-powered multisite RCT designs.

### Meta-analyses of CAMS

#### Therapeutic assessment meta-analysis

Poston and Hanson (35) empirically demonstrated that the CAMS-based SSF assessment functions as a “therapeutic assessment” (i.e., a clinical assessment experience that contributes to both improved treatment process and outcomes). The authors conducted a rigorous meta-analysis of effect sizes from 17 published studies of different psychological assessments (e.g., assessments of self-evaluation or alcohol use among others). Similar to other assessments in the study that met the criteria to be considered a “therapeutic assessment,” this meta-analysis indicated that CAMS assessment is indeed therapeutic in that it provides personalized, collaborative, and highly involving test feedback which contributes to positive clinically meaningful treatment effects. By definition, therapeutic assessments have positive and clinically meaningful effects on treatment, including the improvement of the treatment process. This meta-analysis provides a different sort of validation of CAMS with a strong effect size for CAMS as a therapeutic assessment.

#### Meta-analysis of nine CAMS trials

A significant development related to CAMS clinical trial research occurred when Swift and colleagues (36) conducted a meta-analysis of nine CAMS clinical trials (16, 18, 20–23, 37–39). In comparison to control treatments, results showed that CAMS significantly reduced the primary outcome measure of suicidal ideation and secondary outcome measures showed positive and significant CAMS effects on overall symptom distress, hope/hopelessness, and treatment acceptability. There was a non-significant impact in terms of CAMS on the primary outcome measures of suicide attempts/self-harm and for secondary measures of cost-effectiveness and other suicide-related correlates (e.g., self-esteem and resilience). While overall all outcomes in this meta-analysis favored CAMS when compared to control care, more data are needed to see if non-significant trending findings might reach significance. Beyond the noted primary and secondary outcomes, there were no significant differences between the use of CAMS with White versus non-White samples. Moreover, there was no apparent “publication bias” or “allegiance effect” linked to the developer of CAMS (DAJ). Given these results, Swift et al. concluded that CAMS is “well supported” as a clinical intervention for suicidal ideation as per the criteria of the Centers for Disease Control and Prevention (which is the designation for the highest level of empirical support). It should be noted that five of the previously discussed CAMS RCTs were used in the Swift et al. study and four additional non-randomized clinical trials of CAMS were also included in the nine-study meta-analysis.

### CAMS training research

Pisani and colleagues (40) noted that training in CAMS has been recognized as one of only a handful of suicide-specific professional training approaches at the national level. Schuberg and colleagues (41) conducted an unpublished study of 165 CAMS-trained Veterans Affairs mental health providers and found significant (*p* < 0.05) before and after training differences related to decreasing clinician anxiety about working with suicidal risk and that training increased clinician confidence in the skills of assessing and treating suicidal risk. There were significant pre–post positive training findings related to clinicians’ perceptions about increasing their skills related to forming an alliance with a suicidal patient, increasing patient motivation, and conducting safety planning. Most of these significant CAMS training pre–post effects were sustained in a 3-month follow-up assessment with a subset of the original sample (*n* = 36).

LoParo, Florez, Valentine, and Lamis (42) investigated a handful of suicide-focused trainings in the U.S. state of Georgia. The team studied 137 mental health providers who were varyingly trained in four suicide-focused approaches, namely, CAMS, DBT, Assessing and Managing Suicide Risk (AMSR), and Question, Persuade, Refer (QPR). The results of this investigation showed that the CAMS training—in comparison to other trainings—was significantly more impactful in terms of the outcome variable of “clinical confidence” to work with suicidal risk (i.e., CAMS instilled more confidence in providers than other trainings).

An online survey of 120 mental health practitioners conducted by Crowley, Arnkoff, Glass, and Jobes (43) found moderate to high self-reported adherence to the CAMS therapeutic philosophy, which was comparable to other studies gauging the impact of suicide-focused training. Participants further self-reported relatively high adherence to CAMS practice, which was higher than the findings on adherence to interventions for other issues.

Finally, there is an unpublished doctoral dissertation that investigated the “CAMS Integrated Training Model” (ITM) offered to 116 mental health professionals (44). The ITM included a) didactic training using a 3-hour online clinical demonstration course, b) 1 day of experiential role-play training of the model, and c) six to eight consultation phone or video coaching sessions. The study showed good support for the ITM approach to professional training in CAMS. Statistical analyses of self-report assessments showed statistically significant improvements in clinician attitude, knowledge about CAMS and suicide, and the acquisition of clinical skills to use CAMS effectively to assess and treat suicidal risk following the training.

## Dialectical Behavior Therapy

DBT was originally developed for chronically suicidal and/or self-injuring women meeting the criteria for borderline personality disorder (BPD). At the time of development in the 1970s and 1980s, Linehan reports being most interested in addressing the chronic suicidality found in individuals with severe problems regulating their emotional experiences (45). However, she also reports that she was told that in order to get funding from the National Institutes of Health, she needed to study chronic suicidality within a specific psychological disorder. She chose the condition of BPD because it is woefully underfunded and misunderstood. Since the original trial of DBT was published (46), it quickly became used and studied with a much broader target population than BPD and is often considered a transdiagnostic treatment.

Standard comprehensive outpatient DBT involves four modes of treatment delivered by a treatment team. These modes are individual therapy, skills training (usually conducted in a group format), as-needed intersession consultation between therapist and client, and therapist consultation team. These modes of treatment are delivered following a guiding set of assumptions and principles. Examples of assumptions that guide DBT delivery are as follows: “Clients are doing the best they can,” “The lives of suicidal clients with borderline personality disorder are unbearable as they are currently being lived,” and “Therapists treating suicidal clients need support.” Examples of principles that inform DBT delivery are a set target hierarchy that guides therapists on what to address in any given session, the need to dialectically balance acceptance and change in strategies and problem solutions, and the importance of behavioral specificity in problem definition and solution. Individual therapy sessions are delivered by the primary therapist who is also responsible for suicide risk management, with assistance from consultation team members. Skills training sessions focus on teaching skills in four domains (mindfulness, interpersonal effectiveness, emotion regulation, and distress tolerance). Intersession phone coaching calls are designed to assist with skills generalization though are often also utilized to address suicide crisis. Therapist consultation team meetings function to improve therapist adherence to the DBT model, provide support, and reduce burnout.

One reason that DBT is often now considered a transdiagnostic treatment for individuals at risk for suicide is because its target hierarchy is informed by behaviors, in order of importance, rather than diagnostic “symptoms.” The target hierarchy that informs DBT prioritizes “life-threatening behaviors.” Life-threatening behaviors include suicide urges and crisis behaviors, suicide attempts, non-suicidal self-injury, and suicide ideation. This target hierarchy guides the therapist to focus on life-threatening behaviors as the top priority even if there is a myriad of other problems that the client is experiencing or prefers to discuss. The therapist applies behavioral principles (e.g., behavioral assessment and problem-solving), acceptance principles (via validation strategies and mindfulness practice), and dialectical principles (via modeling of a dialectical worldview which encourages “both and” thinking and use of dialectical strategies when polarization occurs) to affect change in target behaviors. Throughout the treatment, there is additional emphasis on getting an active commitment to not kill oneself and to stay in treatment.

## Empirical support for DBT

There have been dozens of RCTs and quasi-experimental studies on DBT over the past 30+ years. Many of these studies have been focused on populations with suicide risk or behavior, but many have focused on other populations/target problems. For this review, there will be a focus on key RCTs that study the full model (all four modes) of DBT, meta-analyses, and training research and only include studies for which suicidal behavior was a primary focus. An exhaustive review of all DBT studies is beyond the scope and focus of our integrative discussion.

### Key randomized controlled trials of DBT

#### Original DBT trial

Linehan et al. (46) conducted the first RCT of DBT. In this study, 44 women with borderline personality disorder and “parasuicidal^1^” behavior were randomized to receive 1 year of either DBT or TAU. The primary outcomes were self-harm behaviors [suicide attempts and non-suicidal self-injury (NSSI)], the severity of those behaviors, and the frequency and duration of psychiatric hospitalizations. Results indicated that DBT had greater decreases in all these outcomes compared to TAU (refer to **Table 2** for the included DBT RCTs).

**Table 2:** The included DBT RCTs.

Authors	*N*	Sample/setting	Study inclusion	Main outcomes
Linehan et al., 1991 (46)	44	Community sample, Seattle, WA	≥2 SA or NSSI behaviors in the past 2 years	Greater reductions in SA and NSSI severity, reductions in frequency and duration of psychiatric hospitalizations
Koons et al., 2001 (47)	28	Durham VA Medical Center outpatients, Durham, NC	Women veterans meeting the criteria for BPD^1^	Decreased SI, hopelessness, depression, and anger expression
Linehan et al., 2006 (48)	101	University of Washington clinic and community practice outpatients, Seattle, WA	Women meeting criteria for BPD, ≥2 SA/NSSI behaviors in the past 5 years (≥1 in 8 weeks prior to enrollment)	Reductions in SA, fewer psychiatric hospitalizations, fewer days of psychiatric hospitalization for SI, lower medical risk for NSSI behaviors
Linehan et al., 2015 (49)	99	University of Washington clinic and community practice outpatients, Seattle, WA	Women meeting criteria for BPD, ≥2 SA/NSSI behaviors in the past 5 years (≥1 in 8 weeks prior to enrollment), SA in the past year	Reductions in frequency and medical severity of SAs, reduced SI, increased use of crisis services; conditions with DBT skills training components saw more improvements in NSSI frequency
McCauley et al., 2018 (50)	173	Adolescent outpatients from four academic medical centers across Seattle, WA and Los Angeles, CA	≥1 lifetime SA, elevated past month SI, ≥3 NSSI episodes (≥1 in 12 weeks prior to enrollment)	Reductions in SAs, NSSI episodes, and self-harm episodes during and after treatment; decreased rates of self-harm episodes through 1 year of follow-up
McMain et al., 2009 (51)	180	Outpatients at the Centre for Addiction and Mental Health and St. Michael’s Hospital, Toronto, CA	Meeting criteria for BPD, ≥2 SA/NSSI behaviors in the past 5 years (≥1 in 8 weeks prior to enrollment)	Both GPM and DBT saw reductions in frequency and severity of SAs, NSSI, ED visits, and psychiatric hospital stays; however, there was no difference between conditions

1 Suicidal behavior was not an inclusion criterion; however, 75% endorsed ≥1 instance of self-injury.

#### First DBT trial with an independent investigator

Early trials of DBT included Linehan as the investigator as well as trial therapist. In order to establish the effects of DBT as wide ranging, positive effects of the treatment must be determined by at least two different investigators [e.g (52)]. The first published RCT of DBT by an investigator other than Linehan was by Koons and colleagues (47). In this study, 28 women seen through the Durham VA Medical Center and who met the criteria for BPD were randomized to either DBT or TAU. Although history of suicidal behavior was not an inclusion criterion for this study, 75% of the participants endorsed at least one intentional self-injury episode. Results indicated that participants randomized to DBT had significantly greater decreases in suicidal ideation, hopelessness, depression, and anger expression. In addition, there was a trend for a statistically significant reduction in the number of intentional self-injury behaviors and hospitalizations for participants in the DBT condition; however, these were not statistically different from individuals in the TAU condition. Both of these earlier trials, while important in the initial establishment of DBT’s potential, had small sample sizes and limited statistical power to detect differences. More studies with larger samples were then conducted.

#### DBT compared to treatment-by-experts

In order to demonstrate DBT’s efficacy compared to a more stringent control condition and with a larger sample, Linehan et al. (48) randomized 101 women who met the criteria for BPD and were considered high risk for the suicide group. This was operationalized as inclusion criteria of at least two suicide attempts or self-injuries in the past 5 years, with at least one episode in the 8 weeks prior to enrolling. The treatment conditions were either DBT or “Community Treatment by Experts” (CTBE) which was developed specifically for this study. CTBE was conducted by clinicians who had been nominated by their community as experts in treating clients with difficult behaviors. CTBE was also designed to match DBT in terms of availability of treatment and supervision. Participants were provided up to 1 year of treatment and then assessed for 1 year of follow-up.

Results from this study indicated that DBT outperformed CTBE on most primary outcomes, including the number of suicide attempts (individuals in DBT were half as likely to make an attempt than in CTBE), fewer psychiatric hospitalizations, fewer days of hospitalization for suicide ideation, and lower medical risk for self-injurious episodes. This study was designed to overcome limitations of prior research on DBT as well as criticisms that DBT’s effects were based solely on general factors of expert psychotherapy.

#### Component analysis study

In an effort to determine whether the full model of DBT is necessary or whether a less intensive (and therefore less expensive) treatment would be as efficacious, Linehan and colleagues conducted a component analysis study (49). The three treatment conditions were full model DBT, DBT skills training “only” which included weekly skills training plus non-DBT individual therapy, and DBT individual therapy “only” which included weekly individual therapy and non-DBT activity-based support group. All three treatments were designed to control for the amount of therapy received as well as for peer supervision/consultation. In addition, therapists in all three conditions were trained in the DBT crisis management protocol, given the high-risk sample. Participants included 99 women who met the criteria for BPD and had similar suicide risk inclusion criteria to Linehan et al. (48).

Results from the component analysis study indicated that all conditions result in similar improvements in frequency and medical severity of suicide attempts, suicide ideation, and use of crisis services. The two conditions with DBT skills training had statistically greater improvements in frequency of NSSI acts compared to the DBT individual therapy only condition. These results suggested that a variety of DBT interventions could be useful for reducing suicidal behavior. Complementing prior research that indicated that skills use is an important mediator of outcomes in DBT trials (53), interventions that included DBT skills training may be more effective than interventions without.

#### DBT for adolescents at risk for suicide

DBT for adolescents (DBT-A) had been developed by Miller, Rathus, and Linehan (54) and studied in a series of uncontrolled trials with generally positive effects. This multisite study (50), conducted in Seattle and Los Angeles, randomized 173 adolescents ages 12–18 (95% female subjects) to DBT or individual and group supportive therapy. Inclusion criteria were at least one lifetime suicide attempt; elevated past-month suicidal ideation; at least three self-injury episodes, with 1 in the 12 weeks before enrollment; and three or more borderline personality disorder criteria. In the DBT-A condition, as per the manual, skills training sessions included at least one parent/caretaker for each child with the purpose of teaching parents the skills as well.

The primary outcomes for this 6-month treatment trial were suicide attempts, NSSI episodes, and total self-harm episodes. Results indicated that DBT had significantly better outcomes on all of these variables at post-treatment. Specifically, adolescents in DBT were less likely to have a suicide attempt, NSSI episode, or self-harm behavior during treatment, and rates of self-harm decreased through a 1-year follow-up period. However, these treatment differences disappeared by 12 months as both groups improved over time. This was the first adolescent RCT of DBT that demonstrated DBT’s efficacy at reducing suicide attempts in this high-risk group.

#### Largest RCT of DBT

To date, the largest RCT of DBT has been conducted by McMain and colleagues (51) in Canada with 180 participants randomly assigned to treatment. This study was largely designed as a replication study of Linehan (48) with similar inclusion criteria. One exception is that the study included men and women, though men comprised just 14% of the sample. The control condition in this study was General Psychiatric Management (GPM) which was based on the APA Practice Guideline for the Treatment of Patients with Borderline Personality Disorder (2001) and was comprised of case management, psychodynamically informed therapy, and medication management delivered by expert psychiatrists.

In contrast to the other RCTs reviewed here, the McMain et al. (51) study did not find differences between the two conditions on primary outcomes. Both groups demonstrated significant reductions on frequency and severity of suicide attempts and non-suicidal self-injury as well as reductions in emergency room visits and psychiatric hospital days. This study indicated that individuals with BPD and suicidal behavior can benefit from structured, well-specified treatment.

### Meta-analyses of DBT

A number of meta-analyses have been conducted on DBT. Included here is a summary on two that focused on comprehensive DBT’s effects on suicidal behavior specifically.

#### Meta-analysis on 18 DBT trials

DeCou et al. (55) conducted a meta-analysis on 18 trials of DBT that assessed self-injury and suicidality, including suicide attempts, NSSI, suicidal ideation, and access of psychiatric crisis services. The meta-analysis only included studies that compared DBT to TAU or wait-list control, thus removing any studies with more active control conditions. Results indicated that DBT reduced self-directed violence (suicide attempts and NSSI) and reduced the frequency of psychiatric crisis services. This meta-analysis did not find an effect of DBT with regard to suicide ideation.

#### Meta-analysis on DBT for adolescents in 21 studies

Kothgassner et al. (56) included 21 studies in this meta-analysis of DBT outcomes for adolescents ages 12–19. The meta-analysis included only studies that reported outcomes for self-injury and/or suicide ideation and included individuals with a history of at least one suicide attempt or self-injury episode. The sample of studies was comprised of five RCTs, three controlled clinical trials, and 13 pre–post evaluations. Results indicated that DBT demonstrated small to moderate effects for reducing self-injury and suicide ideation, compared to control conditions.

### DBT training research

Given the inherent complexity of DBT, there has also been interest in the development and evaluation of training methods. The gold standard method of training clinicians in DBT is the “Intensive” model which typically takes the form of two 5-day trainings spaced approximately 6 months apart that is attended by teams of clinicians (as opposed to clinicians attending on their own). A study on the adoption of DBT following intensive training with 52 teams found that 75% of the teams adopted all four DBT modes by 8 months after training (57). The adoption of more modes of DBT was predicted by lower training needs and program needs, fewer bachelor’s-level clinicians, and greater prior DBT experience. This study suggests that programs/teams with more resources and fewer stressors can more readily adopt the full model of DBT.

Another method of training that has received some attention is the training of clinicians while still in graduate programs. Given that DBT is a complex treatment that requires significant knowledge with principles of behaviorism, it could be that teaching clinicians early in training through their university training clinics may be an optimal time to learn the treatment. Indeed, in a study conducted at the Dialectical Behavior Therapy Clinic at Rutgers University (DBT-RU), it was found that therapist trainees delivering DBT could achieve similar outcomes to a benchmarked gold-standard RCT (58). The growing demands for a workforce that can deliver DBT with competence and adherence to the model suggest that targeting individuals while still in graduate school may be effective for increasing access to DBT.

## Clinical trials using both CAMS and DBT

As noted, there have been RCT efforts to investigate CAMS and DBT together. The aforementioned Danish DiaS RCT was an initial effort to compare DBT and CAMS within a superiority RCT with a population that would be optimally suited for DBT because the patients in the trial had made suicide attempts and had borderline personality disorder features (21). In this study, both treatments were proven effective in decreasing primary outcome variables of suicide attempts and self-harm with data trending in favor of CAMS (even while the “dose” of CAMS was much less, e.g., 10 sessions once per week vs. 16 sessions of twice per week meetings).

As previously noted, Pistorello and colleagues (32) pursued a feasibility SMART design to explore eight sessions of CAMS vs. eight sessions of TAU in stage 1. For those who insufficiently respond to stage 1, a second stage 2 randomization occurred to either more CAMS (up to 16 weeks) or DBT (up to 16 weeks). Funded by the NIMH, there was not enough grant support to fully populate the SMART design and only 12 clients were randomized to stage 2 which was too small a sample to analyze. Phase 1 results only were thus published showing that CAMS was significantly more effective than TAU in terms of the primary outcome of suicidal ideation and the secondary outcome of depression. However, in an interesting moderator analysis, TAU was generally more effective than CAMS for patients in this trial who had multiple suicide attempt histories and borderline features. This finding is consistent with the extensive evidence base highlighting the therapeutic superiority of DBT with more chronic, dysregulated, multiple attempting individuals [e.g (12, 55, 59)]. An alternative moderator finding was that, in clients who were “newer” to suicidal ideation with no attempt history or borderline features, CAMS significantly decreased a secondary outcome measure of hopelessness when compared to TAU. In other words, the notion “one size does not fit all” seems to emerge from these preliminary RCT findings [refer to (60)].

In an effort to fully test the SMART design, the National Institute of Mental Health funded a multisite study using four university counseling centers in the United States (University of Oregon, University of Nevada–Reno, Duke University, and Rutgers University). At the time of this writing, the “Comprehensive Adaptive Multisite Prevention of University Student Suicide” (CAMPUS trial) is in the final year of data collection in which 480 students will be randomized to stage 1 of eight sessions of CAMS vs. eight sessions of TAU, followed by stage 2 randomization for non-responders to either eight more sessions of CAMS or eight sessions of Counseling Center DBT (CC-DBT). This ambitious trial was directly impacted by the COVID-19 pandemic in 2020 which resulted in two feasibility trials in which training and delivery of all study treatments were performed online with notable success (61).

## The use of CAMS and DBT within contemporary clinical practice

Beyond the clinical research focus, there are examples of the routine use of CAMS and DBT within day-to-day contemporary clinical care. At the anecdotal level, we have seen success with the sequencing of care wherein CAMS might be used initially to stabilize a patient in relation to suicide from which they can progress into intensive DBT. Even within the earliest RCT of CAMS, some of the best outcome cases were the ones where patients received eight sessions of CAMS and then referred to intensive DBT within the DBT-rich environment of Seattle, Washington (20).

### The Hope Institute

The clearest example of the combined clinical use of CAMS and DBT is The Hope Institute (THI). Founded by Derek Lee in Perrysburg Ohio, THI is an outpatient clinical setting where adults and youth who are suicidal are seen with a singular clinical goal of *stabilization.* Given the known limits of emergency department care and inpatient care (11), THI offers a major and compelling alternative model to the routine use of these more restrictive medically oriented settings. The THI model embraces a next-day-appointment (NDA) approach to clinical care and patients can be seen intensively (up to four times/week). All patient in the THI receive CAMS that might be supplemented by the DBT skills group. In terms of clinical THI outcomes, youth have been stabilized in 5.5 weeks, and adults are typically stabilized in 6 weeks. Patients are able to maintain their stability until they are engaged in ongoing outpatient care (62).

Based on the success of the Perrysburg setting, three new Hope Institutes are in various stages of development and use. One is providing care in the Chandler School District of Arizona (seeing high school and middle school teens who are suicidal), another is seeing patients at a children’s hospital in Georgia, and a third clinic is now being initiated in Boulder, Colorado. There is interest in establishing more such clinics offering a major alternative approach to working with suicidal risk on an outpatient basis. Based on the Perrysburg model, the system can be self-sustaining and increased fees can be negotiated with healthcare plans secondary to the savings from not relying on expensive restrictive medical care. Staff morale at THI is high and clinicians receive better salaries than they would otherwise receive in community mental health agencies. Relying on team support and clinical consultation, THI is doing important and innovative evidence-based clinical care with adult and youth patient at risk for suicide.

## Discussion

Suicide is a major public health and mental health challenge around the world. There are many costs associated with suicidal suffering for both patients and their loved ones. It is well known that mental health providers are challenged by patients who have suicidal thoughts and behaviors, and in the United States, there is fear of malpractice liability should there be an adverse clinical outcome. Given these considerations, there is a strong need for clinical interventions that will effectively treat suicidal risk, and there has been considerable progress over the past three decades to this end. There are now a handful of suicide-related clinical treatment approaches proven to be effective with the support of randomized clinical trials that reliably replicate therapeutic outcomes (with independent validations). We have thus reviewed two clinical approaches that rise to the highest level of clinical trial rigor. CAMS and DBT now have extensive RCT and meta-analytic support, and there is also further research support for training in these respective approaches. We have also noted emerging developments in the use of CAMS and DBT within routine clinical practice.

Going forward, the next steps for further increasing the use of CAMS and DBT will be shaped and ultimately defined by current ongoing clinical trial research and ever-evolving clinical practices within contemporary mental healthcare. As discussed by Jobes (11), the advent of the “988 Suicide & Crisis Lifeline” in the United States is now creating an increasing awareness related to emotional crises and suicidal risk. As governments around the world endeavor to enhance crisis services with crisis lines and centers, there is increasing awareness that we must go beyond an acute crisis-only approach with a clear need to *treat* what causes suicidality (63, 64).

Moreover, as discussed by Jobes and Chalker (60), there will likely never be a “one size fits all” treatment approach for suicidal risk across different populations and settings. Accordingly, we contend that the adherent use of CAMS and DBT separately, sequentially, or perhaps even together offers a compelling and complementary approach for clinically addressing suicidal risk. Along these lines, Ronald Kessler’s notion of “precision treatment rules” might one day enable us to rely on machine learning-generated algorithms to route appropriate patients to proven evidence-based treatments for whom they are optimally suited for effective care as well as saving treatment costs (65). We would finally note that the best approach to treating suicidal risk—and decreasing liability—is to use proven clinical practices that are shown to effectively treat suicidal risk. Given the proven efficacy of CAMS and DBT, there is considerable promise in being able to provide both of these clinical approaches for effectively treating the spectrum of suicidal risk (from acute suicidal ideation to chronic states with a history of multiple suicide attempts), thereby reducing suicidal suffering and related behaviors across patient populations and clinical settings.

## Author contributions

DJ: Writing – original draft, Writing – review & editing. SR: Writing – original draft, Writing – review & editing.

## Footnotes

1 At the time of Linehan’s original study and book publication, the term “parasuicidal” was used as an umbrella term to include behaviors with suicidal intent (e.g., suicide attempts) and behaviors with non-suicidal intent (e.g., NSSI). This term has since fallen out of favor.
